# Antioxidant Activities of Extract and Fractions from the Hypocotyls of the Mangrove Plant *Kandelia candel*

**DOI:** 10.3390/ijms11104080

**Published:** 2010-10-21

**Authors:** Shu-Dong Wei, Hai-Chao Zhou, Yi-Ming Lin

**Affiliations:** Key Laboratory of the Ministry of Education for Coastal and Wetland Ecosystems, School of Life Sciences, Xiamen University, Xiamen 361005, China

**Keywords:** Kandelia candel, hypocotyl, antioxidant activity, MALDI-TOF MS, HPLC

## Abstract

The antioxidant activities of 70% acetone extract (70% AE) from the hypocotyls of the mangrove plant *Kandelia candel* and its fractions of petroleum ether (PF), ethyl acetate (EF), water (WF), and the LF (WF fraction further purified through a Sephadex LH-20 column), were investigated by the 1,1-diphenyl-2-picrylhydrazyl (DPPH) free radical scavenging and ferric reducing/antioxidant power (FRAP) assays. The results showed that all the extract and fractions possessed potent antioxidant activity. There was a significant linear correlation between the total phenolics concentration and the ferric reducing power or free radical scavenging activity of the extract and fractions. Among the extract and fractions, the LF fraction exhibits the best antioxidant performance. The MALDT-TOF MS and HPLC analyses revealed that the phenolic compounds associated with the antioxidant activity of the LF fraction contains a large number of procyanidins and a small amount of prodelphinidins, and the epicatechin is the main extension unit.

## 1. Introduction

Reactive oxygen species (ROS), including superoxide radicals, hydroxyl radicals, singlet oxygen and hydrogen peroxide, are often generated as by-products of biological reactions or from exogenous factors [1]. Increasing evidence has suggested that many human diseases, such as cancer, cardiovascular disease, and neurodegenerative disorders, are the results of the oxidative damage by reactive oxygen species [2,3]. The antioxidants are molecules that mainly decelerate or prevent the oxidation reaction *in vitro* and *in vivo* by terminating the oxidation chain reaction [4]. The application of antioxidants in pharmacology is valuable to improve current treatments for diseases.

In recent years, there has been a great interest in finding natural antioxidants from plant materials to replace synthetic antioxidants, which are being restricted due to their carcinogenicity [5]. Numerous crude extracts and pure natural compounds from plants were reported to have antioxidant and radical scavenging activities [6–8]. Within the antioxidant compounds, flavonoids and phenolics, with a large distribution in nature, have been studied more comprehensively [9–11].

Mangroves are a diverse group of trees that grow in intertidal tropical forests. In mangrove species, phenolics are abundant components, which prevent damage from herbivores [12,13], but they also exhibit a diversity of other biological activities of historic and potential importance to humans [14]. Mangrove extracts have been used for diverse medicinal purposes and have a variety of antibacterial, antiherpetic and antihelminthic activities [15,16]. The extracts of some mangrove species indicate significant antioxidant activity [17,18]. *Kandelia candel* (Rhizophoraceae) is mostly widely distributed in the tropical and subtropical coastlines of China. According to a previous study, phenolics are important components in the leaf extract of *K. candel* and show excellent antioxidant activities [19]. The hypocotyls of *K. candel* also have high phenolics levels [20]. Therefore, the *K. candel* hypocotyls may be a good candidate for further development as an antioxidant remedy. In this study, we investigated the antioxidant activities of the 70% acetone extract and its fractions of *K. candel* hypocotyls for the first time, and identified the active compounds by matrix-assisted laser desorption/ionization time-of-flight mass spectrometry (MALDI-TOF MS) and reversed phase high performance liquid chromatography (HPLC) analyses.

## 2. Results and Discussion

### 2.1. DPPH Radical Scavenging Activity

**Figure 1:**
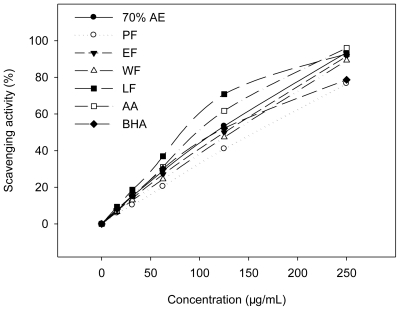
DPPH radical scavenging activities of AA (ascorbic acid), BHA and the extract/fractions of *K. candel* hypocotyls at different concentrations.

DPPH is one of the compounds that has a proton free radical with a characteristic absorption, which decreases significantly on exposure to proton radical scavengers [21]. The DPPH radical scavenging by antioxidants is attributable to their hydrogen donating ability [22]. The DPPH assay has been widely accepted as a tool for estimating free radical scavenging activities of antioxidants [19,23–27]. Figure 1 illustrates a significant decrease in the concentration of DPPH radical due to the scavenging ability of the extract/fractions and standards (ascorbic acid and BHA).

The quality of the antioxidants in the extract/fractions was determined by the IC_50_ values (the concentration with scavenging activity of 50%) (Table 1). A low IC_50_ value indicates strong antioxidant activity in a sample. The lowest IC_50_ value of the LF (87.20 ± 1.01 μg/mL) indicated that this fraction exhibited the highest radical scavenging effect. The DPPH radical scavenging activity was found to be in the order: LF > ascorbic acid > BHA ≈ 70% AE > EF > WF > PF.

### 2.2. Ferric Reducing Antioxidant Power (FRAP)

**Figure 2:**
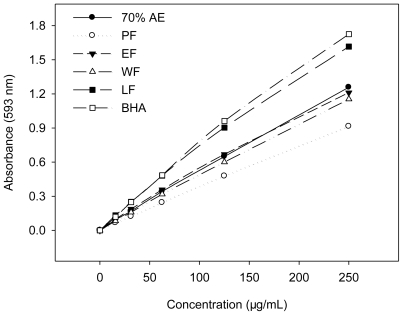
Ferric reducing power of BHA and the extract/fractions of *K. candel* hypocotyls at different concentrations.

The reduction capacity of a compound may serve as a significant indicator of its potential antioxidant activity [28]. The FRAP assay treats the antioxidants contained in the samples as reductants in a redox-linked colorimetric reaction and the value reflects the reducing power of the antioxidants [29]. A higher absorbance corresponds to a higher ferric reducing power. In the present study, the BHA and extract/fractions showed increased ferric reducing power with the increased concentration (Figure 2).

The FRAP value was expressed in ascorbic acid equivalents to determine the antioxidant ability of the samples. The FRAP was found to be 4.18 ± 0.36, 2.99 ± 0.27, 4.39 ± 0.17, 3.69 ± 0.04, 5.91 ± 0.23, and 5.28 ± 0.11 mmol AAE/g for 70% AE, PF, EF, WF, LF, and BHA, respectively (Table 1). In accordance with the findings from the DPPH assay, the LF had the highest antioxidant ability. In brief, the reducing power of extract/fractions and standard exhibited the descending order: LF > BHA > 70% AE ≈ EF > WF > PF.

### 2.3. The Total Phenolics Concentration

Plant phenolics possess the ability to scavenge both active oxygen species and electrophiles [30]. Many plant phenolic compounds, including flavonoids, tannins and phenolic acid, exhibit a strong antioxidant activity [31,32]. The total phenolics concentration in the extract/fractions of the hypocotyls of *K. candel* is shown in Table 2.

**Figure 3:**
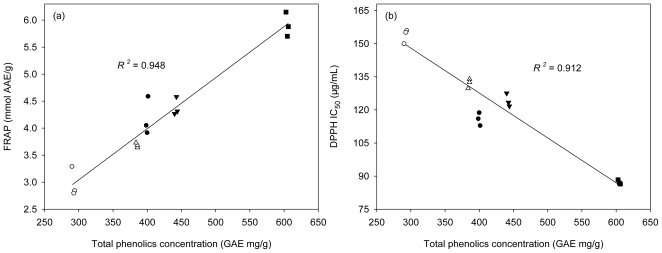
Correlation between the total phenolics concentration and the ferric reducing power (**A**); the total phenolics concentration and the free radical scavenging activity (**B**) of the extract/fractions of the hypocotyls of *K. candel*. Symbols: black circles = 70% AE, white circles = PF, black triangles = EF, white triangles = WF, and black quadrangle = LF.

LF had the highest concentration of total phenolics, followed by EF, 70% AE, WF, and then PF. A significant liner correlation was found between the total phenolics concentration and the ferric reducing power (*R^2^* = 0.948) or the total phenolics concentration and free radical scavenging activity (*R^2^* = 0.912) (Figure 3). Some previous studies also had the same results [33–35]. Phenolics concentration of *K. candel* hypocotyls was responsible for the antioxidant activity.

### 2.4. MALDI-TOF MS Analysis

MALDI-TOF MS is ideally suited for characterizing polyflavonoid tannin oligomers [36–38] and is considered the mass spectrometric method of choice for analysis of tannins, which exhibit large structural heterogeneity [39]. When MALDI-TOF is used to characterize tannins, the selection of the appropriate matrix and cationization reagent is very important. Flamini [40] and Behrens *et al*. [41] reported that DHB as a matrix leads to the best analytical conditions for the detection of procyanidins in reflectron mode to provide the broadest mass range with the least background noise. Xiang *et al*. [42] found that for various cationizing agents in the presence of DHB matrix for MALDI-TOF MS analysis of condensed tannins, only Cs^+^ affect the intensity of the signals on the MALDI-TOF mass spectrum [42]. Using MALDI-TOF with deionization and selection of Cs^+^ as the cationization reagent, higher tannin polymers were observed [43].

**Figure 4:**
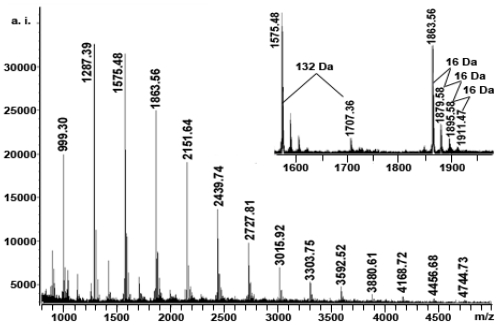
MALDI-TOF positive ion reflectron mode mass spectrum of the condensed tannins from the last fraction (LF).

Figure 4 shows the MALDI-TOF mass spectrum of the polymeric tannin mixtures from the last fraction (LF), recorded as Cs^+^ adducts in the positive ion reflectron mode and showing a series of repeating procyanidin polymers. The displayed magnification demonstrates the good resolution of the spectrum. The results indicated that condensed tannins from the last fraction (LF) are characterized by mass spectra with a series of peaks with distances of 288 Da, corresponding to a mass difference of one catechin/epicatechin between each polymer (Table 3). Therefore, prolongation of condensed tannins is due to addition of catechin/epicatechin monomers, extending up to hexadecamers.

In addition to the predicted homopolyflavan-3-ol mass series mentioned above, each DP had a subset of masses 16, 32, 48 Da higher (Figure 4 and Table 3). These masses can be explained by heteropolymers of repeating flavan-3-ol units containing an additional hydroxyl group (.16 Da) at the position 5′ of the B-ring. Given the absolute masses corresponding to each peak, it was further suggested that this condensed tannin contains a large number of procyanidins and a small amount of prodelphinidins.

On the basis of the structures described by Krueger *et al*. [44], an equation was formulated to predict heteropolyflavan-3-ols of a higher DP (Table 3). The equation is *M* = 290 + 288*a* + 304*b* + 133, where *M* is calculated mass, 290 is the molecular weight of the terminal catechin/epicatechin unit, *a* is the degree of polymerization contributed by the catechin/epicatechin extending unit, *b* is the degree of polymerization contributed by the gallocatechin/epigallocatechin extending unit, and 133 is the weight of cesium. Application of this equation to the experimentally obtained data revealed the presence of a series of condensed tannins consisting of well-resolved polymers. The broad peaks in this spectrum indicated, however, that there is a large structural heterogeneity within DP.

Each peak of the condensed tannins was also followed by mass signals at a distance of 132 Da (Figure 4), which was most likely added by one arabinoside group at the heterocyclic C-ring or an additional one Cs^+^ and loss of a proton. No series of compounds that are 2 Da multiples lower than those described peaks for heteropolyflavan-3-ols were detected, so A-type interflavan ether linkage does not occur between adjacent flavan-3-ol subunits. For the first time, the structure of condensed tannins from the hypocotyls of *K. candel* was successfully characterized by MALDI-TOF MS.

### 2.5. Thiolysis with Cysteamine Followed by RP-HPLC Analysis

**Figure 5:**
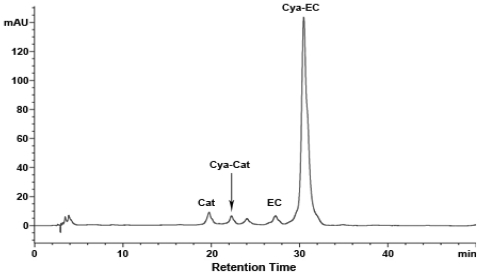
Reversed phase HPLC chromatograms of the tannins from the last fraction (LF) degraded in the presence of cysteamine; Cat, (+)-catechin; EC, (−)-epicatechin; Cya-Cat, 4β-(2-aminoethylthio) catechin; Cya-EC, 4β-(2-aminoethylthio) epicatechin.

Depolymerization reactions in the presence of nucleophiles are often used in the structural analysis of condensed tannins [18]. To further investigate whether the condensed tannins from the last fraction (LF) are composed of catechin and epicatechin, depolymerization through thiolysis reaction was carried out by following standard procedures using cysteamine, which was preferred to toluene-*a*-thiol as it is more user-friendly and much less toxic [45]. The reaction mixture was analyzed by HPLC (Figure 5). In agreement with the MALDI-TOF MS results, the last fraction (LF) consists primarily of procyanidins. The major product observed was the 4β-(2-aminoethylthio) epicatechin (Cya-EC) along with a small amount of (+)-catechin (Cat), (−)-epicatechin (EC), and 4β-(2-aminoethylthio) catechin (Cya-Cat). This result suggests that there are significant amounts of epicatechin extension units in the last fraction (LF).

## 3. Experimental Section

### 3.1. Chemicals and Materials

The solvents acetone, petroleum ether, ethyl acetate and methanol were of analytical reagent (AR) purity grade. The trifluoroacetic acid (TFA) and acetonitrile used for the analysis were of HPLC grade. 1,1-Diphenyl-2-picrylhydrazyl (DPPH), 2,4,6-tripyridyl-S-triazine (TPTZ), cysteamine hydrochloride, ascorbic acid, butylated hydroxyanisole (BHA), cesium chloride, and gallic acid were purchased from Aldrich (U.S.). (−)-Epicatechin (EC), (+)-catechin (Cat) were purchased from Sigma (U.S.). Sephadex LH-20 was purchased from Amersham (U.S.). The hypocotyls of *K. candel* were collected from Zhangjiang River Estuary Mangrove National Natural Reserves (117°24′E, 23°55′N), Yunxiao, Fujian province, China, and immediately freeze dried and ground.

### 3.2. Preparation of Samples

Freeze-dried hypocotyls (15 g) of *K. candel* were extracted with 150 mL 70% acetone and kept for 24 h at room temperature. The extract was then centrifuged at 3000 g for 15 min and collected. The same procedure was repeated three times. The collected extracts were combined, concentrated in a rotary evaporator, and then lyophilized. The final yield of 70% acetone extract (70% AE) was 4.5 g. From the 4.5 g of dried extract, 3 g was fractionated successively with petroleum ether, ethyl acetate, and water to yield soluble fractions of petroleum ether (PF, 0.21 g), ethyl acetate (EF, 0.39 g), and water (WF, 2.36 g). The water fraction (2 g) containing mainly polymers was further purified through a Sephadex LH-20 column. The column was first eluted with methanol:water (1:1) until the eluent turned colorless and then with acetone:water (7:3, 500 mL). The acetone was removed under reduced pressure, and the resulting residue was lyophilized to give the last fraction containing the purified polymeric tannins (LF, 0.89 g), which was further analyzed by MALDI-TOF mass spectrometry and thiolysis.

### 3.3. DPPH Radical Scavenging Activity

The free radical scavenging activities of the samples on the DPPH radical were measured using the method described by Brand-Williams *et al*. [46]. A 0.1 mL of various concentrations of each freeze-dried sample at different concentrations (15.63–250 μg/mL) was added to 3.9 mL of DPPH solution (25 mg/L in methanolic solution). An equal amount of methanol and DPPH served as control. After the mixture was shaken and left at room temperature for 30 min, the absorbance at 517 nm was measured. Lower absorbance of the reaction mixture indicates higher free radical scavenging activity. The IC_50_ value, defined as the amount of antioxidant necessary to decrease the initial DPPH concentration by 50%, was calculated from the results and used for comparison. The capability to scavenge the DPPH radical was calculated by using the following equation:

$$\text{DPPH scavenging effect}(\%)=[({\text{A}}_{1}-{\text{A}}_{2})/{\text{A}}_{1}]\times 100$$

where A_1_ = the absorbance of the control reaction; A_2_ = the absorbance in the presence of the sample. BHA and ascorbic acid were used as standards.

### 3.4. Ferric Reducing/Antioxidant Power (FRAP) Assay

FRAP assay is a simple and reliable colorimetric method commonly used for measuring the total antioxidant capacity [47]. In brief, 3 mL of prepared freshly FRAP reagent was mixed with 0.1 mL of test sample or methanol (for the reagent blank). The FRAP reagent was prepared from 300 mmol/L acetate buffer (pH 3.6), 20 mmol/L ferric chloride and 10 mmol/L TPTZ made up in 40 mmol/L hydrochloric acid. All the above three solutions were mixed together in the ratio of 25:2.5:2.5 (v/v/v). The absorbance of reaction mixture at 593 nm was measured spectrophotometrically after incubation at 25 °C for 10 min. The FRAP values, expressed in mmol ascorbic acid equivalents (AAE)/g dried tannins, were derived from a standard curve.

### 3.5. Determination of Total Phenolics

The amount of total phenolics was determined using the Folin-Ciocalteu method [48]. Briefly, 0.2 mL aliquot of extract was added to a test tube containing 0.3 mL of distilled H_2_O. 0.5 mL of Folin-Ciocalteu reagent and 2.5 mL 20% Na_2_CO_3_ solution were added to the mixture and shaken. After incubation for 40 min at room temperature, the absorbance *versus* a blank was determined at 725 nm. Total phenolics concentrations of extracts were expressed as mg gallic acid equivalents (GAE)/g extract. All samples were analyzed in three replications.

### 3.6. MALDI-TOF MS Analysis

The MALDI-TOF MS spectra were recorded on a Bruker Reflex III instrument (Germany). The irradiation source was a pulsed nitrogen laser with a wavelength of 337 nm, and the duration of the laser pulse was 3 ns. In the positive reflectron mode, an accelerating voltage of 20.0 kV and a reflectron voltage of 23.0 kV were used. 2,5-Dihydroxy benzoic acid (DHB, 10 mg/mL 30% acetone solution) was used as the matrix. The sample solutions (10 mg/mL 30% acetone solution) were mixed with the matrix solution at a volumetric ratio of 1:3. The mixture (1 μL) was spotted to the steel target. Amberlite IRP-64 cation-exchange resin (Sigma-Aldrich, U.S.), equilibrated in deionized water, was used to deionize the analyte-matrix solution thrice. Cesium chloride (1.52 mg/mL) was mixed with the analyte-matrix solution (1:3, v/v) to promote the formation of a single type of ion adduct ([M + Cs]^+^) [42].

### 3.7. Thiolysis of the Condensed Tannins for HPLC Analysis

Thiolysis was carried out according to the method of Torres and Lozano [49]. A condensed tannin solution (4 mg/mL in methanol) was prepared. A sub-sample (50 μL) was placed in a vial and hydrochloric acid in methanol (3.3%, v/v; 50 μL) and cysteamine hydrochloride in methanol (50 mg/mL, 100 μL) were added. The solution was heated at 40 °C for 30 min, and cooled to room temperature. The size and composition of the condensed tannins were estimated from the RP-HPLC analysis of the depolymerised fractions [45]. Briefly, the terminal flavan-3-ols units were released by acid cleavage in the presence of cysteamine, whereas the extension moieties were released as the C4 cysteamine derivatives. Thiolysis reaction media (20 μL) filtrated through a membrane filter with an aperture size of 0.45 μm was analyzed by RP-HPLC.

The high performance liquid chromatograph was an Agilent 1200 system (U.S.) equipped with a diode array detector and a quaternary pump. The thiolysis media were further analyzed using LC/MS (QTRAP 3200, U.S.) with a Hypersil ODS column (4.6 mm × 250 mm, 2.5 μm) (China). The mobile phase was composed of solvent A (0.5% v/v trifluoroacetic acid (TFA) in water) and solvent B (0.5% v/v TFA in acetonitrile). The gradient condition was: 0–5 min, 3% B (isocratic); 5–15 min, 3–9% B (linear gradient); 15–45 min, 9–16% B (linear gradient), 45–60 min, 16–60% B (linear gradient). The column temperature was ambient and the flow-rate was set at 1 mL/min. Detection was at 280 nm and the UV spectra were acquired between 200–600 nm. Degradation products were identified on chromatograms according to their relative retention times and their UV-visible spectra. Each sample analysis was repeated three times.

### 3.8. Statistical Analysis

All data were expressed as means ± standard deviation of three independent determinations. One-way analysis of variance (ANOVA) was used, and the differences were considered to be significant at *P* < 0.05. All statistical analyses were performed with SPSS 13.0 for windows.

## 4. Conclusions

All the extract/fractions from the hypocotyls of *K. candel* showed potent antioxidant activity. Phenolics concentration of *K. candel* hypocotyls was responsible for the antioxidant activity. Extracts from the hypocotyls of *K. candel* might be valuable antioxidant natural sources for both the medical and food industry. Among the extract/fractions, the LF fraction exhibits the best antioxidant performance. The MALDT-TOF MS and HPLC analyses revealed that the phenolic compounds associated with the antioxidant activity of the LF fraction, contains a large number of procyanidins and a small amount of prodelphinidins, and the epicatechin is the main extension unit.

## Figures and Tables

**Table 1:** Antioxidant activities of the 70% acetone extract (70% AE) of *K. candel* hypocotyls and its fractions using the DPPH free radical scavenging assay and the ferric reducing antioxidant power (FRAP) assay.

Extract/fraction/ standard antioxidants	Antioxidant activity	
	IC_50/DPPH_ (μg/mL) a	FRAP (mmol AAE/g) b
70% AE	115.67 ± 2.91d	4.18 ± 0.36c
PF	153.48 ± 3.22a	2.99 ± 0.27e
EF	124.19 ± 3.02c	4.39 ± 0.17c
WF	132.04 ± 2.16b	3.69 ± 0.04d
LF	87.20 ± 1.01f	5.91 ± 0.23a
Ascorbic acid	101.96 ± 1.84e	--
BHA	116.91 ± 0.97d	5.28 ± 0.11b

a The antioxidant activity was evaluated as the content of the test sample required to decrease the absorbance at 517 nm by 50% in comparison to the control;

b FRAP values are expressed in mmol ascorbic acid equivalent/g sample in dry weight; BHA: Butylated hydroxyanisole. Values are expressed as mean of duplicate determinations ± standard deviation; Different letters in the same column show significant differences from each other at *P* < 0.05 level.

**Table 2:** Total phenolics concentration in the extract/fractions of the hypocotyls of *K. candel*.

Extract/fractions	Total phenolics (GAE mg/g extract or fractions)
70% AE	400.43 ± 1.34c
PF	292.75 ± 2.05e
EF	442.21 ± 2.05b
WF	385.02 ± 1.16d
LF	604.63 ± 1.69a

Values are means ± SD of three determinations. Different letters in the same column show significant differences from each other at *P* < 0.05 level.

**Table 3:** MALDI-TOF MS of the condensed tannins from the last fraction (LF).

Polymer	Number of catechin units	Number of Gallocatechin units	Calculated [M + Cs]^+^	Observed [M + Cs]^+^
Trimer	3	0	999	999.30
	2	1	1015	1015.30
	1	2	1031	1031.22

Tetramer	4	0	1287	1287.39
	3	1	1303	1303.39
	2	2	1319	1319.38

Pentamer	5	0	1575	1575.48
	4	1	1591	1591.48
	3	2	1607	1607.49

Hexamer	6	0	1863	1863.56
	5	1	1879	1879.58
	4	2	1895	1895.58
	3	3	1911	1911.47

Heptamer	7	0	2151	2151.64
	6	1	2167	2167.71
	5	2	2183	2183.67
	4	3	2199	2199.59

Octamer	8	0	2439	2439.74
	7	1	2455	2455.75
	6	2	2471	2471.62

Nonamer	9	0	2727	2727.81
	8	1	2743	2743.48
	7	2	2759	2759.67

Decamer	10	0	3015	3015.92
	9	1	3031	3032.92

Undecamer	11	0	3303	3303.75
	10	1	3319	3320.75

Dodecamer	12	0	3591	3592.52
	11	1	3607	3608.53

Tridecamer	13	0	3879	3880.61
	12	1	3895	3896.72

Tetradecamer	14	0	4167	4168.72
	13	1	4183	4184.73

Pentadecamer	15	0	4455	4456.68

Hexadecamer	16	0	4743	4744.73
